# Body mass trajectories and multimorbidity in old age: 12-year results from a population-based study

**DOI:** 10.1016/j.clnu.2021.10.012

**Published:** 2021-10-22

**Authors:** Amaia Calderón-Larrañaga, Xiaonan Hu, Jie Guo, Luigi Ferrucci, Weili Xu, Davide L. Vetrano

**Affiliations:** a Aging Research Center, Department of Neurobiology, Care Sciences and Society, Karolinska Institutet & Stockholm University, Solna, Sweden; b Intramural Research Program, National Institute on Aging, Baltimore, USA

**Keywords:** Body mass index, Multimorbidity, Older adults, Trajectories, Chronic disease

## Abstract

**Background & aims:**

Body weight changes reflect and impact several health conditions in older age, but little is known about its relationship with multimorbidity. We aimed to study the association of long-terms trajectories of body mass index (BMI) with contemporaneous changes in multimorbidity —and multimorbidity type— development in a population-based cohort of older adults.

**Methods:**

Twelve-year BMI trajectories (2001–2013) were identified in subjects aged 60+ years from the Swedish National Study on Aging and Care in Kungsholmen (SNAC-K) using growth mixture models (N = 2189). Information on 60 chronic diseases and multimorbidity was ascertained based on clinical examinations, lab tests, medications, and inpatient and outpatient medical records. Linear mixed models were used to study the association between BMI trajectories and the speed of chronic disease accumulation, in general and by groups of cardiovascular and neuropsychiatric diseases.

**Results:**

Eighty percent of the study population was included in what we defined a *stable* BMI trajectory, 18% in a *slow-decline* trajectory with an accelerated BMI decline from age 78 onwards, and 2% in a *fast-decline* trajectory that reached underweight values before age 85. A significantly higher yearly rate of chronic disease accumulation was observed in the *fast-decline* versus *stable* trajectory (β = 0.221, 95% CI 0.090–0.352) after adjusting the model for age cohort, sex, education and time to death. Subjects in the *slow-decline* trajectory showed a significantly higher yearly rate of cardiovascular disease accumulation (β = 0.016, 95% CI 0.000–0.031); those in the *fast-decline* trajectory showed a faster accumulation of both cardiovascular (β = 0.020, 95% CI −0.025, 0.064) and neuropsychiatric diseases (β = 0.102, 95% CI 0.064 −0.139), even if the former association did not reach statistical significance.

**Conclusion:**

Our results provide further evidence of the importance of carefully monitoring older adults with sustained weight loss, which is an early indicator of accelerated health deterioration, reflected in our study by a faster accumulation of chronic —especially neuropsychiatric— diseases.

## 1. Introduction

A high body mass index (BMI) is a well-recognized risk factor for the development of several chronic diseases [1], eventually leading to a condition of multimorbidity [2]. Moreover, both obesity and multimorbidity are associated with a higher risk of disability, intense healthcare utilization and mortality [3,4]. On the other hand, older adults with overweight show better health status and lower all-cause mortality compared to those with normal or underweight [5]; an observation known as the obesity paradox [6]. This apparent contradiction should be read in light of the bidirectional relationship between body weight and health, and the dynamic body composition changes accompanying aging, which lead to substantial redistributions of fat, muscle and bone mass [7]. Accordingly, BMI changes over time are better predictors of different health-related outcomes such as functional decline [8], dementia [9], and death [10], compared to single-point weight measurements. Yet, for a better understanding of the relationship between BMI and multimorbidity, it should be noted that chronic diseases per se can be major determinants of body weight and body composition changes, as is for example the case for heart disease, cancer, diabetes and several neurodegenerative conditions [11–14].

Previous studies exploring changes in BMI in relation to health outcomes have typically defined BMI change based on pre-established thresholds or have focused exclusively on average changes, which fails to identify distinctive groups of individuals following similar trajectories [15,16]. The assessment of BMI trajectories is therefore central to understand how different weight phenotypes relate to health in old age. There is some evidence showing the existence of distinct BMI trajectories in the older population [17], also in relation to differing risks of functional decline [8,18] and death [19,20]. However, very few investigations have focused on the relationship between body weight changes and multimorbidity, and none of them have differentiated among types of multimorbidity, even if it is known that certain types of e.g. cardiovascular and neuropsychiatric diseases tend to cluster in single individuals majorly contributing to functional impairment and disability [21], and can themselves be expected to trigger different body weight changes over time.

Our aim was to study the association of long-terms trajectories of BMI with contemporaneous changes in multimorbidity —and multimorbidity type— development in a Swedish population-based cohort of older adults. We hypothesized that older people who lose weight at a faster rate are also those showing an accelerated accumulation of chronic diseases. By classifying subjects into mutually exclusive BMI trajectory groups, our study allows a close examination of the heterogeneity in body mass changes throughout old age and enables direct comparison of multimorbidity development across these groups.

## 2. Methods

### 2.1. Study population

The data were gathered from the ongoing longitudinal Swedish National study of Aging and Care in Kungsholmen (SNAC-K) [22]. SNAC-K participants are 60 years and older and live at home or in institutions in the Kungsholmen area of Stockholm, Sweden. At baseline (years 2001–2003), a random selection of participants was performed based on 11 age groups: three younger groups (aged 60, 66 and 72) and eight older groups (aged 78, 81, 84, 87, 90, 93, 96 and 99+), with a participation rate of 73%. The younger cohorts have been followed every six years and the older ones every three years. For this study, the first five waves of data collection were used, covering a maximum of 12 years. SNAC-K was approved by the Swedish Ethical Review Authority, and written informed consent was obtained from participants or their next of kin.

From a baseline study sample of 3363 individuals, 1174 (34.9%) were excluded for this study since they had less than two BMI observations, leaving an analytical sample of 2189 participants (Supplementary Fig. 1). Subjects in the analytical sample were younger, more likely to be male, to have elementary education, and a lower number of chronic diseases and use of drugs, compared with excluded subjects (all p-values <0.001).

### 2.2. Study variables

Weight and height were measured during clinical examinations or self-reported (e.g. 0.8% and 1.0% of baseline weight and height measurements) at all waves. BMI was calculated as subjects’ weight (in kg) divided by the square of body height (in m). Chronic diseases were defined as those with prolonged duration and leaving substantial residual disability or impaired quality of life, or requiring a prolonged period of care, treatment and rehabilitation, and further operationalized according to an expert-based list of 60 chronic conditions [23]. Information on chronic diseases was ascertained based on clinical examinations, lab blood tests, medications, patient history, and inpatient and outpatient medical records. Given their chronic nature, once detected, chronic diseases were considered present in all successive follow-up waves. Apart from looking jointly at all 60 diseases, analyses were further restricted to two groups of chronic conditions: 1) cardiovascular diseases, including ischemic heart disease, heart failure, atrial fibrillation, cerebrovascular disease, cardiac valve diseases, bradycardias or conduction disorders, peripheral vascular disease, and other cardiovascular diseases; and 2) neuropsychiatric diseases, including depression and mood disorders, dementia, neurotic or stress-related and somatoform diseases, migraine and facial pain syndromes, peripheral neuropathy, Parkinson’s disease or parkinsonism, epilepsy, schizophrenia and delusional diseases, multiple sclerosis, other psychiatric or behavioral diseases, and other neurological diseases. Educational level was categorized as elementary, high school and university or higher according to the highest attainment achieved. Information on participants’ date of death was retrieved from the Swedish Death Register.

### 2.3. Statistical analysis

BMI trajectories over the 12-year follow-up were defined using growth mixture models. These models group subjects into different classes based on their similarities in terms of intraindividual BMI change patterns. Age was chosen as the time scale to better account for its confounding effect, and a quadratic slope was included to detect a potential faster decline of BMI at older ages. The number of latent classes was decided through both the Bayesian Information Criterion (BIC) and the Lo-Mendell-Rubin (LMR) likelihood ratio test. Different combinations of random effects were included both for the intercept and the linear slope, and the randomness was either class-specific or class-invariant based on the likelihood ratio test (LRT). The variance of the quadratic slope was assumed to be zero and the residual variances, which were assumed to be equal across classes, were allowed to vary across age groups. Growth mixture models were also run for males and females separately.

Subjects were assigned to the latent class to which they had the highest probability of belonging (e.g. most likely scenario). Linear mixed models were applied to examine the association between the different BMI trajectories and the mean annual change in the number of chronic diseases, overall and for specific groups of diseases. To that end, interactions between follow-up time and BMI trajectories were included as fixed effects in the models. All models were adjusted by baseline age, sex, education and time to death, and further adjusted by other types of chronic diseases when specific groups of diseases (i.e. cardiovascular and neuropsychiatric) were studied. The most stable BMI trajectory (i.e. remaining unchanged and within a normal range over time) was chosen as the reference.

### 2.4. Sensitivity analysis

We examined the influence of missing data due to death or drop-out on the association between BMI trajectories and chronic disease accumulation through joint models, which jointly estimate the parameters of a linear mixed model and a survival model for time to death or drop-out. The survival model included covariates associated with the risk of death or drop-out: sex, age, and baseline BMI. This technique allows the correction of longitudinal estimates by taking attrition into account, given the potential selection bias arising from higher mortality or drop-out in relation to specific BMI trajectories.

Growth mixture models were fitted using MPlus version 8.4; linear mixed models and joint models were performed using Stata version 15.1; and results were processed using R version 3.6.1.

## 3. Results

**Fig. 1.**
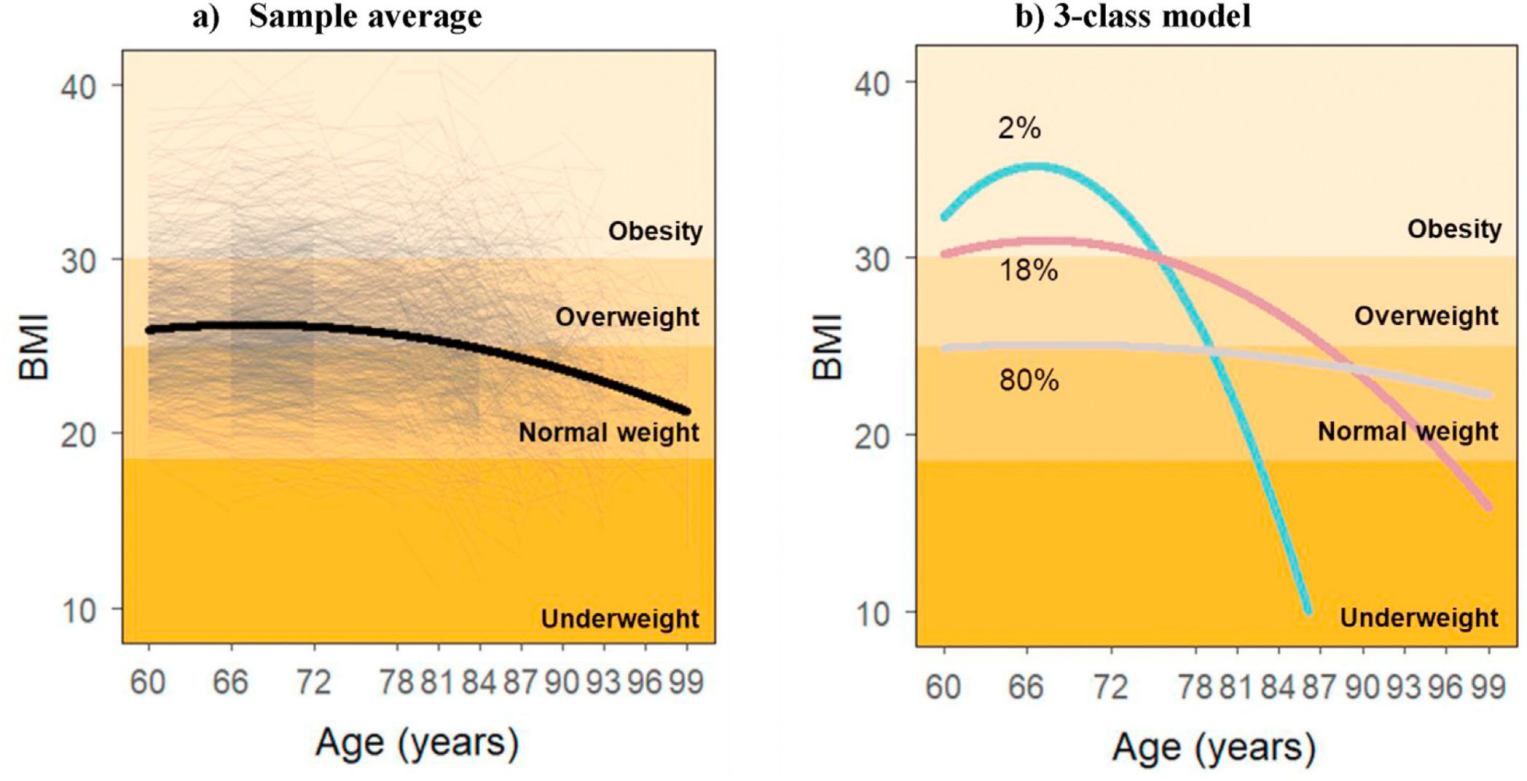
Individual (dotted) and average (solid) BMI trajectories for the total sample.

The study population had a mean age of 72 years, the majority being female (63%) and with above-elementary education (87%) (Table 1). Figure 1 a and b show the BMI sample average and the results from best-fit growth mixture model, respectively, built based on a total of 6717 individual BMI assessments (mean of 3.1 assessments per individual). Eighty percent of the study population was included in what we defined a *stable* trajectory, 18% in a *slow-decline* trajectory, and 2% in a *fast-decline* trajectory. The *stable* trajectory was relatively constant across ages, with an average BMI of 24.9 kg/m^2^ at age 60 and scores that remained within the normal weight range (i.e. 18.5–24.9 kg/m^2^) over time (Supplementary Table 1). Subjects in the *slow-decline* trajectory started with an average BMI of 30.2 kg/m^2^ corresponding to obesity (i.e. ≥30 kg/m^2^) at age 60 and reached underweight values (i.e. <18.5 kg/m^2^) in their 90s. Those in the *fast-decline* trajectory displayed the largest changes among the three trajectories, with a BMI of 32.3 kg/m^2^ at the age of 60 years and a steep decrease leading to underweight values before age 85. Subjects in the *fast-* and *slow-decline* trajectories were more likely to be women, had lower education, presented with a higher number of chronic conditions and used more drugs compared to those in the *stable* BMI trajectory, both in the total sample and within sex groups (Table 1). People with a fast BMI decline were more likely to die throughout the follow-up compared to those in the other two trajectories.

**Fig. 2.**
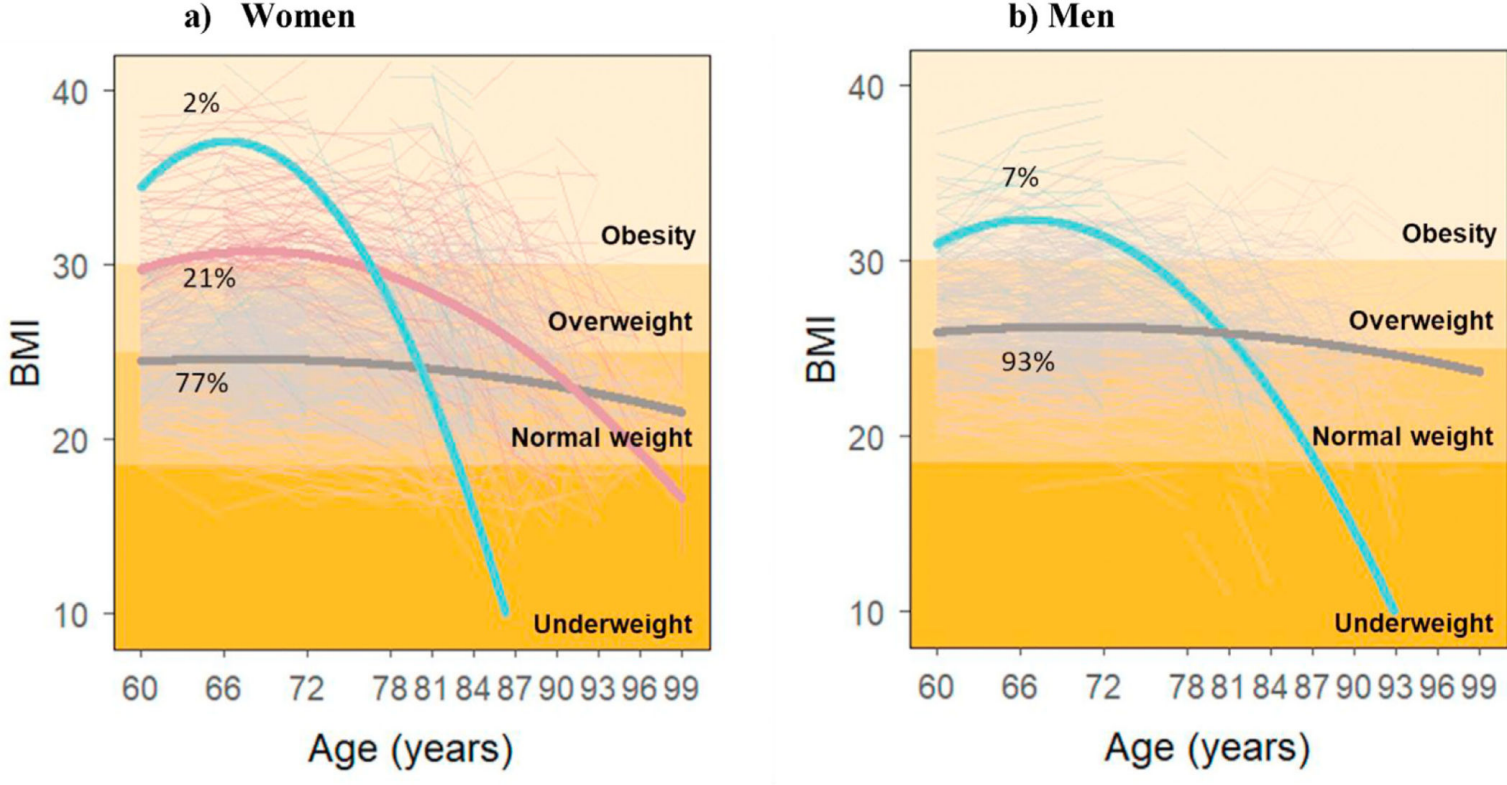
Individual (dotted) and average (solid) BMI trajectories by sex.

Figure 2 a and b show the results from best-fit growth mixture models for women and men separately. While three trajectories were identified for women (N = 1376), only two were identified for men (N = 813). Trajectories identified in women were similar to those in the total sample, with two declining BMI trajectories comprising 23.3% of the population. For men, only one trajectory of declining BMI was identified, including 6.6% of study participants. The parameter estimates and fitting criteria for all these models can be found in Supplementary Tables 1 and 2

**Fig. 3.**
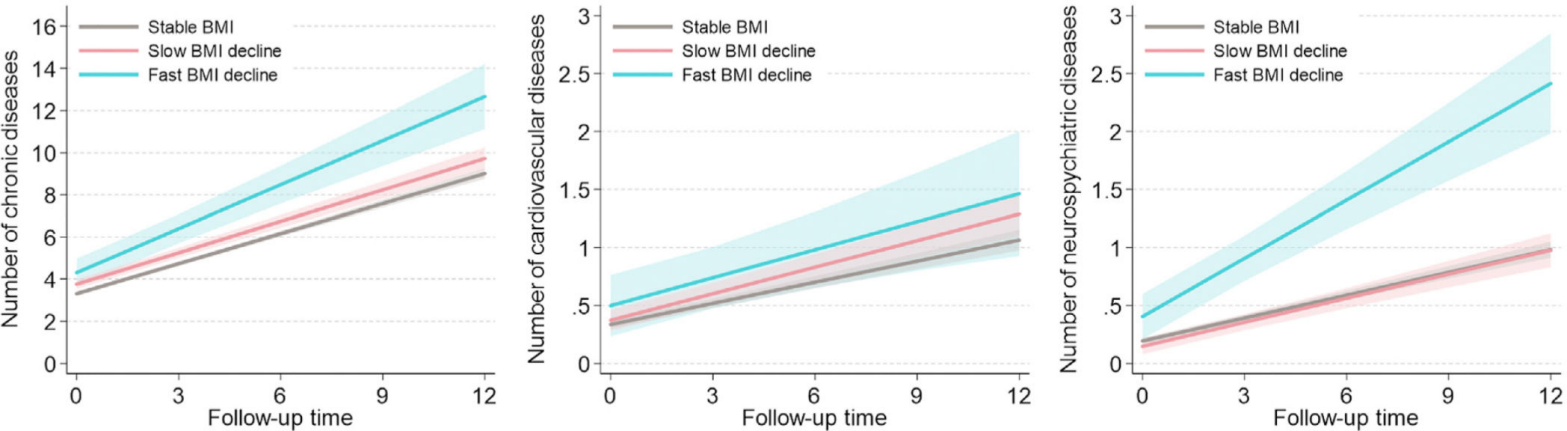
Estimated number or total, cardiovascular and neuropsychiatric chronic diseases over the 12-year follow-up by BMI trajectories. All models are adjusted by education, age at baseline, sex and time to death during follow-up. For the outcomes focusing on system-specific multimorbidity, regressions were further adjusted by the other types of chronic diseases. Cardiovascular diseases include ischemic heart disease, heart failure, atrial fibrillation, cerebrovascular disease, cardiac valve diseases, bradycardias or conduction disorders, peripheral vascular disease, and other cardiovascular diseases. Neuropsychiatric diseases include depression and mood disorders, dementia, neurotic or stress-related and somatoform diseases, migraine and facial pain syndromes, peripheral neuropathy, Parkinson’s disease or parkinsonism, epilepsy, schizophrenia and delusional diseases, multiple sclerosis, other psychiatric or behavioral diseases, and other neurological diseases.

Figure 3 and Supplementary Table 3 show the association between BMI trajectories and the yearly rate of disease accumulation over the 12-year follow-up, for both total and system-specific (i.e. cardiovascular and neuropsychiatric) multimorbidity. For both the *slow-* and *fast-decline* trajectories, a higher number of total and system-specific chronic diseases was observed at baseline compared with the *stable* trajectory, even if some of these differences did not reach statistical significance. At baseline, compared to the *stable* trajectory, subjects in the *fast-decline* trajectory had double the prevalence of cardiovascular diseases such as heart failure (5.8% versus 13.5%), cerebrovascular disease (4.8% versus 10.8%), heart conduction disorders (1.0% versus 2.7%) and peripheral vascular disease (0.9% versus 5.4%), and several neuropsychiatric diseases such as depression and mood disorders (8.2% versus 18.9%), migraine and facial pain syndromes (2.0% versus 5.4%), other psychiatric or behavioral diseases (1.2% versus 5.4%) and Parkinson’s disease or parkinsonism (1.0% versus 2.7%) (Table 2). A significantly higher rate of chronic disease accumulation was observed in the *fast-decline* versus *stable* BMI trajectory (β = 0.221, 95% CI 0.090–0.352) after adjusting the model for age cohort, sex, education and time to death. Subjects in the *slow-decline* trajectory showed a significantly higher yearly rate of cardiovascular disease accumulation (β = 0.016, 95% CI 0.000–0.031). Subjects in the *fast-decline* trajectory showed a faster accumulation of both cardiovascular (β = 0.020, 95% CI −0.025, 0.064) and neuropsychiatric diseases (β = 0.102, 95% CI 0.064–0.139), even if the former association was not statistically significant.

Results from the joint model analyses did not reveal major differences compared to the main analysis (Supplementary Table 4).

## 4. Discussion

In the present study, a faster and earlier body weight decline was associated with a steeper multimorbidity development over 12 years, when compared with a stable BMI over time, even after accounting for the competing risk of death or drop-out from the cohort. This was true for both cardiovascular and, even more strongly, neuropsychiatric diseases. Interestingly, subjects with decreasing BMI trajectories were all obese at baseline. These data provide further rationale for the longitudinal assessment and monitoring of BMI considering its connection with multimorbidity expansion, an acknowledged indicator of an accelerated biological aging [4].

At least two different scenarios can help interpret our findings. First, unintentional weight loss might reflect the acceleration of catabolic processes observed in rapidly aging individuals [24], and a fast decline in body weight may represent the prodromal phase of forthcoming disease development and death [16]. This is in line with the observation that an acceleration in weight loss occurs during the last years of life [10]. Genetic predisposition, malnutrition and unhealthy lifestyles can speed up several biological aging mechanisms (e.g. inflammation, mitochondrial dysfunction) involved in fat and muscle shrinking in older people (i.e. sarcopenia) [6]. Similar mechanisms may link weight loss to a faster accumulation of chronic conditions [25–27]. To note, in our study, the association between fast declining BMI and multimorbidity was strongest for neuropsychiatric chronic diseases including dementia, depression and Parkinson’s disease, which is in consonance with previous studies showing an accelerated weight loss preceding several neurodegenerative conditions and mood disorders as one of their *de facto* subclinical manifestation [28–30].

A second more complex scenario would identify two competing forces at play. Initially, obesity can be framed as a midlife risk factor for disease development in old age. Subsequently, multimorbidity becomes itself the cause of an accelerated weight loss in older ages [31]. The fact that the fastest BMI loss patterns among older adults occur at ranges of weight that correspond to obesity has been previously shown [20,32,33], and may indicate that the BMI with which a person enters into old age is likely to shape future BMI changes and health trajectories in later life [16,18]. Reduced fitness, chronic inflammation and increased insulin resistance seem to be the main factors underlying the negative influence of midlife obesity on disease and multimorbidity development, concerning both cardiovascular [34] and non-cardiovascular diseases [32,35].

Several chronic diseases are associated with an accelerated weight loss, involving both fat and muscle mass, which in extreme cases may derive in cachexia. Chronic diseases are causes per se of increased basal metabolism and systemic inflammation, and are sometimes associated with reduced dietary intake and malnutrition [36]. Some examples include severe heart disease, dementia, Parkinson’s disease and stroke [11,13,37,38]. Involuntary weight loss is a typical feature of the frailty phenotype [39], which is predominantly determined by the burden of chronic diseases [40–42]. Interestingly, it has been observed that unintentional weight loss involves mostly subcutaneous fat, leaving the abdominal fat, which is metabolically active in increasing inflammation and cardiovascular risk, more intact [43]. Older people affected by multimorbidity patterns conformed by neuropsychiatric diseases have an increased risk of incident frailty, mainly due to their detrimental impact on their nutritional status, fatigue, engagement in physical activity, and muscle fitness [44]. Previous research from our group has also shown that cardiovascular and, even more prominently, neuropsychiatric multimorbidity have a clear impact on functional decline and disability [21].

Unlike some previous studies looking at BMI trajectories in the general older population [8,20], we did not find a trajectory of increasing BMI over time in our sample. In the study by Zajacova et al. [20], subjects with stable weight and those with weight gain were similar in terms of initial health and most sociodemographic characteristics, which, according to the authors, may reflect that weight gain during the transition into old age tends to occur among relatively robust individuals. The exceptionally healthy and wealthy nature of the SNAC-K population compared to the rest of Sweden may explain the absence of an increasing BMI trajectory in our study, where almost two-thirds were performing health- or fitness-enhancing physical activity at baseline.

### 4.1. Strengths and limitations

This study’s main strengths include its large size and long follow-up with up to five BMI and chronic disease assessments. Both variables were assessed objectively by trained physicians and nurses and, in the case of chronic diseases, a clinically driven algorithm integrating different sources of data (i.e. physician assessment in SNAC-K, laboratory tests and drug, hospital and outpatient registers) was used, which increases the reliability of the diagnoses.

Several limitations should be noted. First, we could not distinguish between voluntary and involuntary weight loss as we lack this information in SNAC-K, but voluntary weight loss is unlikely to be concomitantly related to negative health outcomes. Thus, weight loss can be assumed to be unintentional. Second, our approach to characterize BMI trajectories does not deal with attrition. Supplementary joint modelling showed no evidence of follow-up bias but suggested that the risk of rapid increase in multimorbidity in older persons with a decreasing BMI trajectory was probably underestimated because of competing mortality or drop-out. Third, the measure of BMI used in the study does not discriminate between lean and fat mass, nor does it allow to differentiate between subcutaneous from abdominal fat, which prevented us from ascertaining the types of changes in body composition that occurred across trajectories. Still, BMI can be easily assessed in clinical practice to monitor weight changes in older adults. Fourth, given the concomitant study of multimorbidity and body mass, as well as the imperfect measure of the latter by only looking at BMI, our data could neither corroborate nor refute the obesity paradox. Fifth, our findings, especially those regarding body mass trajectories, might not be replicable beyond SNAC-K participants, who are fitter and wealthier than the average Swedish older population. Moreover, the selection of the analytical sample, which was younger, more educated, and healthier than the rest of SNAC-K participants, may have led to an underestimation of the associations between morbidity burden and BMI changes. Last, the small size of both declining BMI trajectories might have resulted in low statistical power to detect differences between groups.

## 5. Conclusions

Older people’s BMI and its relationship with health status are not uniform, neither across nor within individuals, which highlights the need for repeated measurements over time in order to better assess their prognosis. Our results provide further evidence of the importance of carefully monitoring older adults with sustained weight loss starting in their 70s. Sustained weight loss may be an early indicator of decline in health, as indicated by the rapid accumulation of chronic –especially neuropsychiatric– diseases. These individuals should receive further clinical workup to understand whether they are affected by sub-clinical conditions that can be treated, therefore preventing the impending deterioration of health. Further studies in larger populations are required for the validation of our findings and their translation into clinical recommendations.

## Supplementary Material

supp material

Appendix A. Supplementary data

Supplementary data to this article can be found online at 10.1016/j.clnu.2021.10.012.

## Figures and Tables

**Table 1:** Baseline descriptive statistics for the total population and by the three BMI trajectories.

	Total sample	Stable BMI			Slow BMI decline			Fast BMI decline		
		Total	Women	Men	Total	Women	Men	Total	Women	Men
n (%)	2189	1853^a^ (84.65)	1130^a^ (82.12)	770^a^ (94.71)	299^a^ (13.66)	220^a^ (15.99)	–	37^a^ (1.69)	26^a^ (1.89)	43^a^ (5.29)
Age (years), mean (SD)	71.65 (9.65)	71.82 (9.77)	72.76 (10.03)	70.13 (9.13)	70.90 (9.07)	72.18 (9.12)	–	69.59 (8.06)	69.24 (7.84)	68.73 (8.19)
Women, n (%)	1376 (62.86)	1151 (62.12)	–	–	200 (66.22)	–	–	27 (72.97)	–	–
Education, n (%)										
Elementary	278 (12.71)	221 (11.93)	145 (12.84)	79 (10.26)	50 (16.72)	42 (19.09)	–	7 (18.92)	4 (15.38)	8 (18.60)
High school	1071 (48.95)	888 (47.95)	586 (51.90)	327 (42.47)	165 (55.18)	124 (56.36)	–	18 (48.65)	13 (50.00)	21 (48.84)
University	839 (38.35)	743 (40.12)	398 (35.25)	364 (47.27)	84 (28.09)	54 (24.55)	–	12 (32.43)	9 (34.62)	14 (32.56)
Chronic diseases, mean (SD)	3.46 (2.16) (2.21)	3.38 (2.17)	3.54 (2.20)	3.28 (2.15)	4.30 (2.38)	4.56 (1.96)	–	3.83 (2.00)	5.35 (2.37)	4.58 (2.41)
Cardiovascular diseases, mean (SD)	0.35 (0.78)	0.35 (0.78)	0.31 (0.74)	0.42 (0.84)	0.36 (0.74)	0.27 (0.65)	–	0.57 (0.96)	0.54 (0.86)	0.58 (0.85)
Neuropsychiatric diseases, mean (SD)	0.21 (0.52)	0.21 (0.52)	0.24 (0.56)	0.18 (0.47)	0.18 (0.44)	0.19 (0.46)	–	0.41 (0.64)	0.46 (0.71)	0.19 (0.39)
Drugs; mean (SD)	3.52 (3.17)	3.40 (3.08)	3.85 (3.15)	2.70 (2.86)	4.08 (3.46)	4.27 (3.29)	–	5.19 (3.94)	6.31 (4.07)	4.28 (4.01)
Dropouts during follow-up, n (%)	320 (14.62)	263 (14.19)	178 (15.75)	95 (12.34)	53 (17.73)	36 (16.36)	–	4 (10.81)	2 (7.69)	9 (20.93)
Deaths during follow-up, n (%)	575 (26.27)	491 (26.50)	285 (25.22)	213 (27.79)	66 (22.07)	46 (20.91)	–	18 (48.65)	13 (50.00)	17 (39.53)

Divergences between total numbers and the final sample size (n = 2189) are due to missing data.

a Reported class counts and proportions are based on individuals’ most likely class membership.

**Table 2:** Prevalence (%) of cardiovascular and neuropsychiatric diseases at baseline and follow-up by the three BMI trajectories.

	Stable BMI			Slow BMI decline			Fast BMI decline		
	Baseline	6 years	12 years	Baseline	6 years	12 years	Baseline	6 years	12 years
**Cardiovascular diseases**									
Ischemic heart disease	11.87	17.16	17.29	15.05	17.96	21.11	13.51	16.13	6.67
Atrial fibrillation	6.42	12.36	15.38	5.69	15.49	20.00	8.11	19.35	26.67
Heart failure	5.83	11.82	13.56	6.02	15.85	23.33	13.51	22.58	33.33
Cerebrovascular disease	4.80	9.84	15.92	5.35	14.08	17.78	10.81	16.13	13.33
Cardiac valve diseases	2.16	5.52	9.83	1.00	3.87	12.22	0.00	3.23	6.67
Other cardiovascular diseases	1.89	6.30	10.01	1.00	5.63	12.22	2.70	6.45	20.00
Bradycardias or conduction disorders	0.97	2.82	3.46	1.34	2.46	2.22	2.70	3.23	0.00
Peripheral vascular disease	0.92	3.36	4.55	0.67	2.46	5.00	5.41	6.45	20.00
**Neuropsychiatric diseases**									
Depression and mood disorders	8.20	13.80	18.20	7.02	11.27	16.11	18.92	32.26	40.00
Neurotic or stress-related and somatoform diseases	3.08	9.54	17.65	1.67	5.63	14.44	5.41	29.03	40.00
Migraine and facial pain syndromes	2.00	3.54	6.64	2.68	4.58	6.11	5.41	6.45	0.00
Other neurological diseases	1.78	3.96	6.82	1.34	3.87	10.56	2.70	12.90	13.33
Dementia	1.73	8.52	10.28	1.34	10.92	15.56	0.00	16.13	20.00
Peripheral neuropathy	1.46	4.44	10.56	2.01	3.17	8.89	0.00	3.23	13.33
Other psychiatric or behavioral diseases	1.19	5.16	6.64	1.67	5.99	10.56	5.41	19.35	26.67
Parkinson’s disease or parkinsonism	1.03	2.64	3.91	0.67	1.76	3.33	2.70	3.23	6.67
Epilepsy	0.54	0.84	0.91	0.00	0.00	2.22	0.00	0.00	0.00
Schizophrenia and delusional diseases	0.38	0.36	0.27	0.00	0.70	0.00	0.00	6.45	0.00
Multiple sclerosis	0.11	0.18	0.09	0.00	0.00	0.00	0.00	0.00	0.00

Prevalence figures at 6- and 12-year follow-ups are calculated including subjects still in the study at each occasion in the denominator.

## Data Availability

Data are from the SNAC-K project, a population-based study on aging and dementia (http://www.snac-k.se/). Access to these original data is available to the research community upon approval by the SNAC-K data management and maintenance committee. Applications for accessing these data can be submitted to Maria Wahlberg (Maria.Wahlberg@ki.se) at the Aging Research Center, Karolinska Institutet.
