# Supplementary material for: Body mass trajectories and multimorbidity in old age: 12-year results from a population-based study
Source: Clin Nutr. Author manuscript; Available in PMC 2025 Jun 10. (PMC12149323; doi:10.1016/j.clnu.2021.10.012)
Supplement: supp material [file NIHMS2023576-supplement-supp_material.docx]

**Body mass trajectories and multimorbidity in old age:**

**12-year results from a population-based study**

Amaia Calderón-Larrañaga, Xiaonan Hu, Jie Guo, Luigi Ferrucci, Weili Xu, Davide L Vetrano

**“OnlineSupplementaryMaterial”**

**Supplementary Figure 1. Flow-chart of the study population.**

**
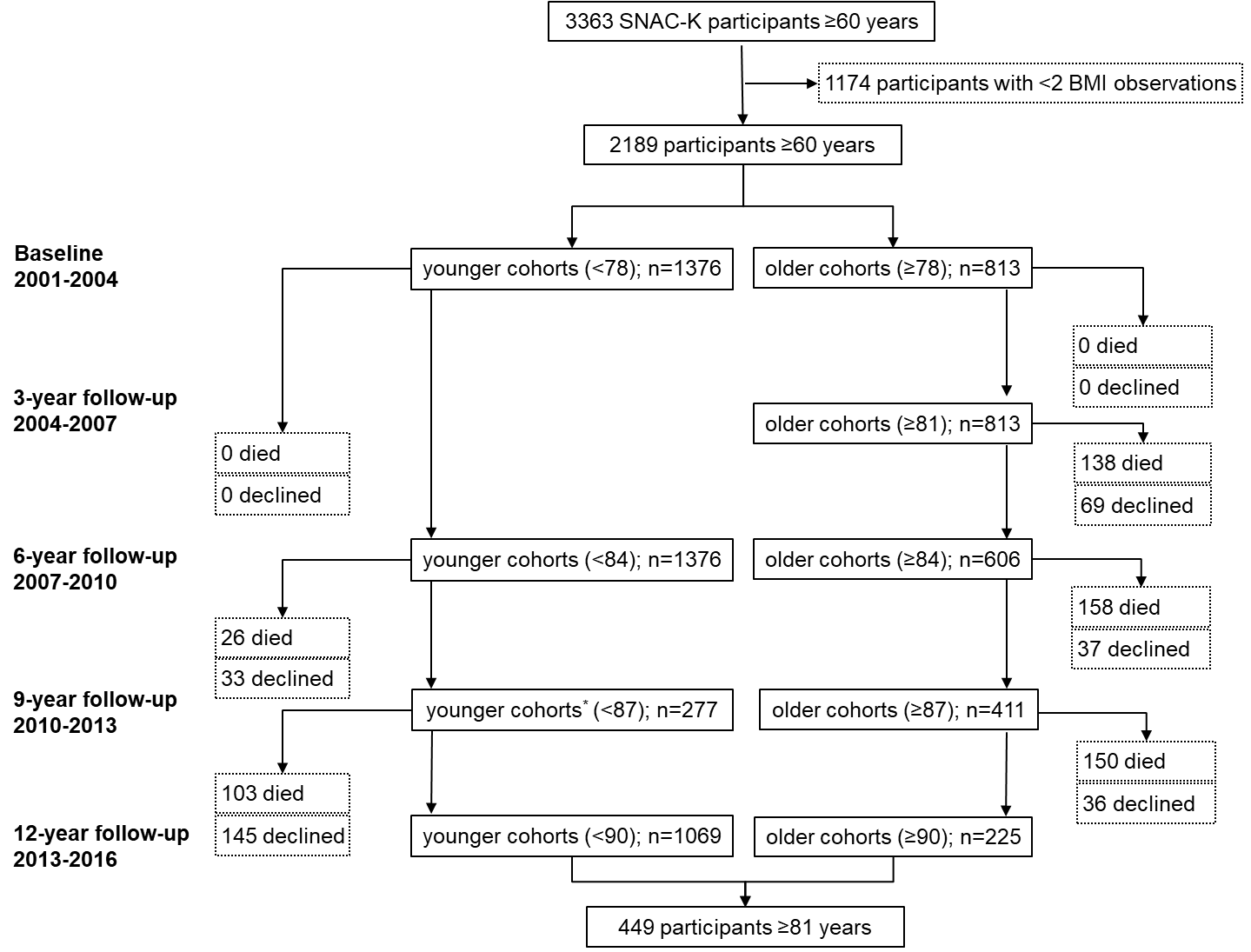
**

**Supplementary Table 1. Parameter estimates for BMI trajectories by latent class for the total sample and by sex.**

|  | **Stable BMI** | **Slow BMI decline** | **Fast BMI decline** |
| --- | --- | --- | --- |
| **Total sample** | | | |
| n (%) | 1853 (84.6%)^#^ | 299 (13.7%)^#^ | 37 (1.7%)^#^ |
| **Fixed effects, mean (SE)** |  | | |
| Intercept (age=60) | 24.90 (0.18) ^***^ | 30.18 (0.72) ^***^ | 32.30 (2.73) ^***^ |
| Linear rate of change | 0.05 (0.01) ^***^ | 0.22 (0.05) ^***^ | 0.87 (0.29) ^***^ |
| Quadratic rate of change | -0.003 (0.000) ^***^ | -0.02 (0.002) ^***^ | -0.07 (0.01) ^***^ |
| **Random effects, mean (SE)** |  |  |  |
| Intercept variance | 6.58 (0.75) ^***^ | 8.37 (1.95) ^*^ | 84.17 (28.35) ^***^ |
| Slope variance | 0.006 (0.002) ^***^ | 0.03 (0.01) ^*^ | 0.68 (0.25) ^***^ |
| **Males** | | | |
| n (%) | 770 (94.7%)^#^ | --- | 43 (5.3%)^#^ |
| **Fixed effects, mean (SE)** |  | | |
| Intercept (age=60) | 25.94 (0.18) ^***^ | --- | 30.98 (0.63) ^***^ |
| Linear rate of change | 0.06 (0.01) ^***^ | --- | 0.42 (0.13) ^***^ |
| Quadratic rate of change | -0.003 (0.000) ^***^ | --- | -0.03 (0.008) ^***^ |
| **Random effects, mean (SE)** |  | | |
| Intercept variance | 7.95 (0.90) ^***^ | --- | 5.67 (2.03) ^***^ |
| Slope variance | 0.008 (0.002) ^***^ | --- | 0.09 (0.03) ^***^ |
| **Females** | | | |
| n (%) | 1130 (82.1%)^#^ | 220 (16.0%)^#^ | 26 (1.9%)^#^ |
| **Fixed effects, mean (SE)** |  | | |
| Intercept (age=60) | 24.45 (0.25) ^***^ | 29.75 (1.43) ^***^ | 34.45 (4.87) ^***^ |
| Linear rate of change | 0.04 (0.03) ^*^ | 0.25 (0.11) ^**^ | 0.84 (0.46) ^*^ |
| Quadratic rate of change | -0.003 (0.001) ^***^ | -0.02 (0.00) ^***^ | -0.07 (0.02) ^***^ |
| **Random effects, mean (SE)** |  | | |
| Intercept variance | 6.32 (1.52) ^***^ | 10.77 (3.35) ^***^ | 94.05 (47.11) ^**^ |
| Slope variance | 0.005 (0.003) ^**^ | 0.03 (0.01) ^***^ | 0.85 (0.34) ^**^ |

^#^Reported class counts and proportions are based on individuals’ most likely class membership.

Significance levels for the coefficients: *p<0.1; **p <0.05; ***p<0.01.

**Supplementary Table 2. Overview of model fit criteria during class enumeration for the total sample and by sex.**

| **# classes** | **# parameters** | **BIC** | **Entropy** | **LMR p-value** | **Smallest class size (%)^*^** |
| --- | --- | --- | --- | --- | --- |
| **Total sample** | | | | | |
| Step1 | | | | | |
| 1 | 14 | 37510.632 | --- | --- | --- |
| 2 | 18 | 34741.248 | 0.830 | 0.000 | 31.9 |
| 3 | 22 | 33371.195 | 0.826 | 0.044 | 11.8 |
| 4 | 26 | 32480.865 | 0.842 | 0.132 | 4.8 |
| Step2 | | | | | |
| 2 | 23 | 30577.332 | 0.841 | --- | 5.7 |
| 3 | 29 | 30458.881 | 0.754 | 0.000 | 1.7 |
| 4 | 35 | 30461.798 | 0.791 | 0.318 | 0.5 |
| **Males** | | | | | |
| Step1 | | | | | |
| 1 | 14 | 13146.073 | --- | --- | --- |
| 2 | 18 | 12052.168 | 0.839 | 0.000 | 35.5 |
| 3 | 22 | 11547.987 | 0.838 | 0.369 | 15.4 |
| 4 | 26 | 11193.909 | 0.858 | 0.004 | 10.8 |
| Step2 | | | | | |
| 2^a^ | 23 | 10551.76 | 0.88 | 0.000 | 5.3 |
| **Females** | | | | | |
| Step1 | | | | | |
| 1 | 14 | 24294.63 | --- | --- | --- |
| 2 | 18 | 22538.361 | 0.84 | 0.002 | 28.6 |
| 3 | 22 | 21629.259 | 0.831 | 0.390 | 9.4 |
| 4 | 26 | 21117.399 | 0.828 | 0.089 | 5.3 |
| Step2 | | | | | |
| 2 | 23 | 19993.24 | 0.814 | 0.05 | 7.2 |
| 3 | 29 | 19897.86 | 0.733 | 0.014 | 1.9 |
| 4^b^ | 35 | 19895.204 | 0.707 | 0.020 | 1.7 |

^*^Reported smallest class sizes are based on individuals’ most likely class membership.

BIC: Bayesian Information Criterion (lower values imply better model fit); LMR: Lo-Mendell-Rubin likelihood ratio test; Entropy: higher values imply better classification quality.

Step 1: selection of number of classes without including any random effects.

Step 2: selection of the best model including random effects, based on the likelihood ratio test (LRT) and the Lo-Mendell Rubin (LMR) test. Step 2 starts from the best model in step1. Once the random effects are included, a final check is conducted to compare among all models with such random effects but with one lower or higher number of classes. It stops when p-value becomes nonsignificant or the model no longer converges with increasing number of classes.

^a^The 3-class solution did not converge. Therefore, we reported the 3-class model with quadratic slope, class-invariant random intercept and random slope.

^b^Although the 4-class solution shows a significant p-value based on the LMR test, the BIC marginally improves. Therefore, we reported the 3-class model with quadratic slope, class-invariant random intercept and random slope.

**Supplementary Table 3. Association between BMI trajectories and: a) baseline number of chronic diseases, b) yearly rate of disease accumulation over the 12-year follow-up.**

|  | **Slow decline vs stable**  **BMI trajectory** | | **Fast decline vs stable**  **BMI trajectory** | |
| --- | --- | --- | --- | --- |
|  | **Cross-sectional association** | **Longitudinal association*** | **Cross-sectional association** | **Longitudinal association*** |
| **Chronic diseases** | 0.449 (0.200, 0.698) | 0.022 (-0.024, 0.067) | 1.000 (0.328, 1.672) | 0.221 (0.090, 0.352) |
| **Cardiovascular diseases** | 0.036 (-0.063, 0.135) | 0.016 (0.000, 0.031) | 0.164 (-0.102, 0.431) | 0.020 (-0.025, 0.064) |
| **Neuropsychiatric diseases** | -0.046 (-0.119, 0.026) | 0.003 (-0.009, 0.016) | 0.210 (0.014, 0.407) | 0.102 (0.064, 0.139) |

*Beta coefficient represent the interaction between BMI trajectories and follow-up time in years.

All regressions are adjusted by education, age cohort, sex and time to death during follow-up. For the outcomes focusing on system-specific multimorbidity, regressions were further adjusted by the other types of chronic diseases.

Cardiovascular diseases include ischemic heart disease, heart failure, atrial fibrillation, cerebrovascular disease, cardiac valve diseases, bradycardias or conduction disorders, peripheral vascular disease, other cardiovascular diseases.

Neuropsychiatric diseases include depression and mood disorders, dementia, neurotic or stress-related and somatoform diseases, migraine and facial pain syndromes, peripheral neuropathy, Parkinson’s disease or parkinsonism, epilepsy, schizophrenia and delusional diseases, multiple sclerosis, other psychiatric or behavioral diseases, other neurological diseases.

**Supplementary Table 4. Association between BMI trajectories and: a) baseline number of chronic diseases, b) yearly rate of disease accumulation over the 12-year follow-up. Results from the joint model.**

|  | **Slow decline vs stable**  **BMI trajectory** | | **Fast decline vs stable**  **BMI trajectory** | |
| --- | --- | --- | --- | --- |
|  | **Cross-sectional association** | **Longitudinal association*** | **Cross-sectional association** | **Longitudinal association*** |
| **Chronic diseases** | 0.391 (0.148, 0.635) | 0.023 (-0.021, 0.067) | 1.354 (0.697, 2.012) | 0.223 (0.094, 0.352) |
| **Cardiovascular diseases** | 0.011 (-0.085, 0.107) | 0.015 (0.000, 0.030) | 0.272 (0.013, 0.531) | 0.027 (-0.017, 0.071) |
| **Neuropsychiatric diseases** | -0.063 (-0.132, 0.005) | 0.004 (-0.009, 0.016 | 0.258 (0.072, 0.444) | 0.108 (0.071, 0.144) |

*Beta coefficient represent the interaction between BMI trajectories and follow-up time in years.

All regressions are adjusted by education, age cohort, sex and time to death during follow-up. For the outcomes focusing on system-specific multimorbidity, regressions were further adjusted by the other types of chronic diseases.

Cardiovascular diseases include ischemic heart disease, heart failure, atrial fibrillation, cerebrovascular disease, cardiac valve diseases, bradycardias or conduction disorders, peripheral vascular disease, other cardiovascular diseases.

Neuropsychiatric diseases include depression and mood disorders, dementia, neurotic or stress-related and somatoform diseases, migraine and facial pain syndromes, peripheral neuropathy, Parkinson’s disease or parkinsonism, epilepsy, schizophrenia and delusional diseases, multiple sclerosis, other psychiatric or behavioral diseases, other neurological diseases.
